# The role of magnetic resonance imaging in the diagnosis and prognostic evaluation of fetuses with congenital diaphragmatic hernia

**DOI:** 10.1007/s00431-022-04540-6

**Published:** 2022-07-07

**Authors:** Ilaria Amodeo, Irene Borzani, Genny Raffaeli, Nicola Persico, Giacomo Simeone Amelio, Silvia Gulden, Mariarosa Colnaghi, Eduardo Villamor, Fabio Mosca, Giacomo Cavallaro

**Affiliations:** 1 Neonatal Intensive Care Unit, Fondazione IRCCS Ca’ Granda Ospedale Maggiore Policlinico, Via Della Commenda 12, 20122 Milan, Italy; 2 Pediatric Radiology Unit, Fondazione IRCCS Ca’ Granda Ospedale Maggiore Policlinico, Milan, Italy; 3 Department of Clinical Sciences and Community Health, Università Degli Studi Di Milano, Milan, Italy; 4 Department of Obstetrics and Gynecology, Fondazione IRCCS Ca’ Granda, Ospedale Maggiore Policlinico, Milan, Italy; 5 Department of Pediatrics, School for Oncology and Reproduction (GROW), Maastricht University Medical Center, University of Maastricht, MUMC+), Maastricht, the Netherlands

**Keywords:** Congenital diaphragmatic hernia, Liver herniation percentage, Liver-to-thoracic volume ratio, Observed/expected total fetal lung volume, Total fetal lung volume, Mediastinal shift angle, Pulmonary hypertension, Survival

## Abstract

In recent years, magnetic resonance imaging (MRI) has largely increased our knowledge and predictive accuracy of congenital diaphragmatic hernia (CDH) in the fetus. Thanks to its technical advantages, better anatomical definition, and superiority in fetal lung volume estimation, fetal MRI has been demonstrated to be superior to 2D and 3D ultrasound alone in CDH diagnosis and outcome prediction. This is of crucial importance for prenatal counseling, risk stratification, and decision-making approach. Furthermore, several quantitative and qualitative parameters can be evaluated simultaneously, which have been associated with survival, postnatal course severity, and long-term morbidity.

*Conclusion*: Fetal MRI will further strengthen its role in the near future, but it is necessary to reach a consensus on indications, methodology, and data interpretation. In addition, it is required data integration from different imaging modalities and clinical courses, especially for predicting postnatal pulmonary hypertension. This would lead to a comprehensive prognostic assessment.

**Table:** 

**What is Known:** *•* *MRI plays a key role in evaluating the fetal lung in patients with CDH.* • *Prognostic assessment of CDH is challenging, and advanced imaging is crucial for a complete prenatal assessment and counseling.*	
**What is New:** *•* *Fetal MRI has strengthened its role over ultrasound due to its technical advantages, better anatomical definition, superior fetal lung volume estimation, and outcome prediction.* • *Imaging and clinical data integration is the most desirable strategy and may provide new MRI applications and future research opportunities.*

**What is Known:**

*•* *MRI plays a key role in evaluating the fetal lung in patients with CDH.*

• *Prognostic assessment of CDH is challenging, and advanced imaging is crucial for a complete prenatal assessment and counseling.*

**What is New:**

*•* *Fetal MRI has strengthened its role over ultrasound due to its technical advantages, better anatomical definition, superior fetal lung volume estimation, and outcome prediction.*

• *Imaging and clinical data integration is the most desirable strategy and may provide new MRI applications and future research opportunities.*

## Magnetic resonance imaging and the fetal lung

Magnetic resonance imaging (MRI) plays a key role in evaluating congenital anomalies affecting the fetal lung, especially when lung hypoplasia is suspected, as occurs in severe oligohydramnios, skeletal dysplasia, lung masses, and congenital diaphragmatic hernia (CDH). In addition, it allows the evaluation of the extent of the anomaly and the amount of normal residual parenchyma [1].

CDH represents a rare congenital malformation affecting 1:3000 live births, characterized by variable degrees of pulmonary hypoplasia and pulmonary hypertension, representing the two most important determinants of the patient’s prognosis [2–4]. Mortality can range from more than 90% in extreme CDH to less than 10% in mild forms. In addition, there is a significant morbidity in survivors [5, 6].

Advanced imaging is crucial for a complete prenatal assessment and parental counseling. Together with genetic testing and ultrasound (US) examination, fetal lung MRI delineates an accurate anatomical picture and contributes to an individualized prediction of disease severity and prognosis. This is essential to identify candidates for fetal intervention and provide the most accurate prognostic and therapeutic information to parents [1, 7, 8].

This review points out the increasing role of fetal MRI in the prenatal assessment and prognostic prediction of patients with CDH and its advantages compared to fetal lung US. We discuss the main quantitative and qualitative prognostic parameters, their association with fetal-neonatal outcomes, and possible future MRI applications.

## Technical aspects and advantages

In CDH, fetal MRI enhances prenatal evaluation through high anatomic specificity of the diaphragmatic defect, hernia location, content, and alteration in other fetal organs [9, 10]. In contrast to US, MRI is not limited by maternal body habitus, fetal position, and amniotic fluid volume [1]. It is less user-dependent and shows excellent repeatability [8, 11]. Since the lung is largely composed of water, it contrasts well against the heart’s darker signal, mediastinum, and liver on T2 sequences [12]. It allows the visualization of both ipsilateral and contralateral lung, with a complete evaluation of the total fetal lung volume (TFLV). In addition, it provides a more accurate liver position than US, since US is limited by the similar echogenicity of the liver and lung [8, 13]. Compressive effects on surrounding structures can also be better evaluated [14]. Due to these favorable aspects, several studies have demonstrated that fetal MRI is superior to 2D and 3D US in diagnosing CDH and outcome prediction [15–19].

The patients can be safely imaged on a 1.5/3 Tesla system using a multichannel cardiac or torso coil, with the fetal region of interest (ROI) within the center of the coil to have an optimum signal [20]. Adequate patient preparation is essential to make the mother feel comfortable and reduce stress and motion artifacts. The mother is positioned supine or on her left side to prevent inferior vena cava syndrome, using pillows and foam pads to maximize patient comfort and immobilization. Other facilitations such as patient coaching, the presence of the partner, or listening to music can be helpful. The mother is entered feet-first to minimize claustrophobia [21]. In addition, fast MR sequences decrease scanning time and allow rapid fetal imaging, which reduces the likelihood of maternal and fetal motion artifacts. At the same time, post-processing approaches further improve image quality [22]. No intravascular contrast agent is generally administered. Usually, a fetal MRI study takes 30–45 min, depending on fetal movements, with a minimum of 15 min [14]. In this way, maternal–fetal sedation can be successfully avoided. However, sedation of the mother does not provide a significant advantage in reducing fetal motion artifacts, requires observation and monitoring after MRI examination, and can adversely affect the developing fetal brain. For these reasons, this practice is rarely used to date [21, 23–26].

Ultrafast T2-weighted sequences, such as single-shot fast spin-echo (SS-FSE), half-Fourier single-shot turbo spin-echo (HASTE), steady-state free precession (SSFP), or True fast imaging with steady precession (FISP), are regarded as the mainstay of fetal MRI and are usually acquired on the three planes of the fetal body. Additional sequences, such as T1-weighted fast gradient-echo images, fast field echo (FFE), fast low angle shot (FLASH), diffusion-weighted images (DWI), or echoplanar imaging may provide further information, especially in the fetal body [27, 28].

## MRI evaluation and clinical implications

### Quantitative parameters

#### Fetal lung volume

**Fig. 1:**
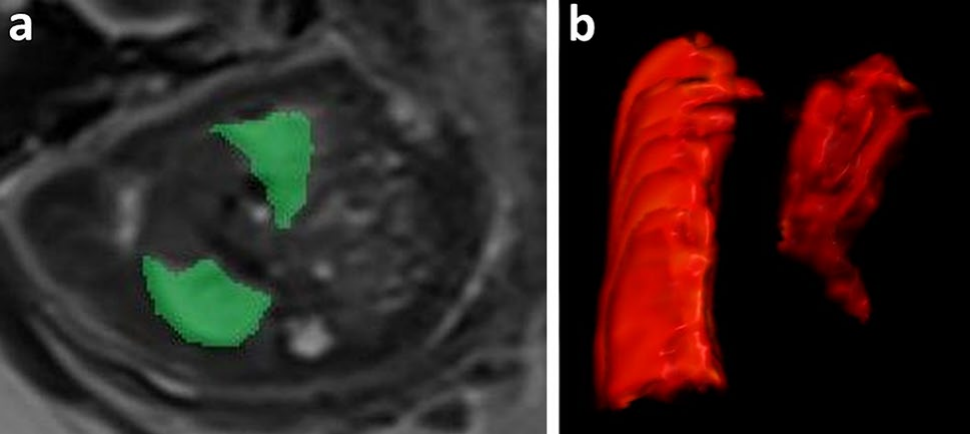
Total fetal lung volume (TFLV) measurement. **a** T2 HASTE axial image showing the lung segmentation methods tracing the region of interest (green) around the left and right lung on each MRI slice. **b** 3D volume rendering reconstruction of the TFLV, obtained by summing each slice area, multiplied by the slice thickness

Improving the stratification of fetuses aims to identify a small subgroup with the most significant benefit from prenatal intervention and to guide clinicians and parents in decision-making during pregnancy. Fetal MRI is the reference technique for fetal lung volume evaluation as an indirect estimation of lung hypoplasia [29–31]. The most common method of measuring lung volumes is based on independently tracing the region of interest around the left and right lung on each MRI slice, excluding the main vessels of the pulmonary hila. The sum of each slice area, multiplied by the slice thickness, gives the TFLV (Fig. 1) [29]. T2-fast sequences (SS-FSE/HASTE) are the most used, with the whole thorax covered on a single acquisition. However, several studies have shown that the lung measurements are independent of sequence, plan, and section thickness. Therefore, the sequence less affected by artifacts can be chosen [31, 32]. In addition, this method shows high reproducibility and excellent inter- and intraobserver agreement [29, 32, 33]. In a recent meta-analysis including four studies assessing the role of TFLV in predicting mortality, the absolute lung volume was markedly reduced in patients with poor prognosis, with a significant overall impact on survival [12].

In order to improve outcome prediction, the observed value of fetal lung volume (oFLV) is usually converted to a percentage of what is expected (eFLV) for a normal fetus of the same gestational age (GA) based on normative data. The two formulas proposed by Rypens et al. and Meyers et al. are the most widely used in clinical practice [29, 34]. The Rypens formula $(eFLV ={0.0033} \times {GA}^{2.86})$ was derived in 2001 from a study that enrolled 336 patients and historically represented the normative reference [29]. Meyers formula (${eFLV=0.000865 \times GA}^{3.254})$ was derived in 2018 from the largest sample size of 665 patients, with the highest proportion of fetuses evaluated at lower GA (167 fetuses at 18–22 weeks of gestation) [34]. A comparison between the two formulas showed an excellent correlation for most gestational ages, but Meyers’ study found significantly lower values of TFLV at 19–22 weeks of gestation. The small number of fetuses and the lack of fetuses < 21 weeks of gestation in Rypens’ study could justify this difference. Therefore, Meyers and coll. suggested using their referential values to avoid potential errors, especially at lower GAs, where prenatal counseling is the most critical. Finally, no differences were found when comparing imaging planes in manual versus semiautomatic methods [34].

In newborns with CDH, the observed/expected total fetal lung volume (o/e TFLV) better correlates with the postnatal outcome than the absolute volume and represents an independent predictor of postnatal mortality and morbidity [35]. There is also increasing evidence that predicting survival with o/e TFLV is more accurate than US estimation of lung size, which does not consider the ipsilateral lung, and could underestimate the actual lung volume [17, 18, 36, 37].

In isolated CDH, o/e TFLV showed good performance in discriminating survival, with a cutoff value of o/e TFLV < 25% associated with most severe forms and < 25% survival rate (Table 1) [12, 17, 38–41]. In addition, the o/e TFLV was shown to predict the need for extracorporeal membrane oxygenation (ECMO) after birth, and the combined evaluation of the lung volumetry and o/e LHR was superior to US alone in predicting the need for ECMO [5, 42–44]. The o/e TFLV was also significantly lower in patients requiring a patch and more prolonged postoperative mechanical ventilation [38].

**Table 1:** Fetal MRI assessment and clinical significance

***TFLV*** ***Expected normal values of TFLV are calculated according to reference formulas:*** ***Rypens et al. ***[29] ${\boldsymbol{eFLV}}=\mathbf{0.0033}\times \boldsymbol{GA}^{2.86}$ ***Meyers et al.*** [34] ${\boldsymbol{eFLV}}=\mathbf{0.000865}\times \boldsymbol{GA}^{3.254}$			
TFLV	*Cut off values*		*Survival reports*
Lee et al. [40]	≤ 20 mL ≥ 40 mL		35% 90%
Neff et al. [39]	5 mL 30 mL		16% 99.6%

***o/e TFLV*** ***It is inversely related to survival: significant improvement in survival is observed for O/E TFLV* > *35%***			
o/e TFLV	*Cutoff values*		*Survival reports*
Alfaraj et al. [41]	< 25% 25–35% > 35%		0% 25% > 75%
Victoria et al. [35]	< 25% 25–35% > 35%		13% 69% 83%
Jani et al. [17]	< 25% 25–35% > 35%		25% 58.6% 80.5%

***PPLV*** ***It is inversely related to survival: significant increase in mortality is observed for PPLV* < *15%***			
PPLV	*Cutoff values*		*Survival reports*
Barnewolt et al. [47]	< 15% > 15%		40% 100%

***Liver position*** ***Assess if the liver is in the normal abdominal position or intrathoracic***			
LiTR	*Cut-off values*		*Survival reports*
Worley et al. [54]	≥ 20% < 20%		14% 87%
%LH	*Cutoff values*		*Survival reports*
	* Severe CDH*	%LH > 21% and O/E TFLV < 32%	47.6%
Ruano et al. [58]	*Moderate CDH*	%LH > 21% and O/E TFLV ≥ 32% or %LH ≤ 21% and O/E TFLV < 32%	80.9%
	* Mild CDH*	%LH ≤ 21% and O/E TFLV ≥ 32%	92.1%

***Stomach position*** ***Assess if the stomach is in the normal abdominal position or intrathoracic***			
Stomach grading	*Clinical significance*		
Nawapun et al. [63]	*Grade 0*	No stomach in the thoracic cavity	Inverse correlation with o/e TFLV
	*Grade 1*	Stomach in the left thoracic cavity	
	*Grade 2*	Less than half of the stomach in the right thoracic cavity	
	*Grade 3*	More than half of the stomach in the right thoracic cavity	
	*Grade 4*	The stomach occupies the entire right side of the thoracic cavity	

***Diaphragm*** ***Assess the position and extent of the diaphragmatic defect***			
DDR	*Cutoff values*		*Surgical approach*
Rygl et al. [67]	< 15 > 15		Primary repair Patch repair

***Mediastinal shift*** ***Assess the shift of mediastinal structures***			
MSA	*Clinical significance*		
Savelli et al. [72]	Inversely associated with survival (suggested cutoff 38.2°)		
Amodeo et al. [73]	Associated with higher intensity of cares		

***Vascular assessment*** ***Assess potential prenatal predictors of severe pulmonary hypertension***			
PPHI and MGI	*Clinical significance*		
Vuletin et al. [110]	PPHI = (left pulmonary artery/length of vermis) × 10 MGI = (right pulmonary artery + left pulmonary artery)/aorta		Inverse correlation with severe PH

***Qualitative evaluation***			
ADC	*Clinical significance*		
Cannie et al. [13]	ADC values deviate from the normal maturational pattern in fetuses with CDH, reflecting the structural differences of the hypoplastic lung		

LLSIR	*Clinical significance*		
Yamoto et al. [118]	Marker of fetal lung maturity and correlates with postnatal survival (o/e LLSIR cutoff value 70)		
Dütemeyer et al. [15]	LLSIR is associated with postnatal survival		

*%LH* liver herniation percentage, *ADC* apparent diffusion coefficient, *CDH* congenital diaphragmatic hernia, *DDR* defect-diaphragmatic ratio, *eFLV* expected fetal lung volume, *GA* gestational age, *LiTR* liver-to-thoracic volume ratio, *LLSIR* lung-to-liver signal intensity ratio, *MGI* McGoon Index,* MSA* mediastinal shift angle, *o/e TFLV* observed/expected total fetal lung volume, *PPHI* prenatal pulmonary hypertension index, *PPLV* percentage of predicted lung volume, *TFLV* total fetal lung volume

In some centers, the TFLV is compared to the predicted lung volume based on fetal body volume rather than gestational age. This could be appropriate if a CDH fetus is also growth-restricted and is expected to have smaller TFLV than the normal growing counterparts, but this practice is less common. The proposed discriminating value for the percentage of the predicted lung volume is 15% [45–48].

Regarding long-term morbidities, several studies demonstrated a strong association between small lung volumes and postnatal development and grading of chronic lung disease (CLD), defined as the need for oxygen supplementation on day 28 after birth, with the best cut of the value of o/e TFLV < 35% [38, 42, 49, 50]. Lung volumetry was also associated with the duration of oxygen supplementation and oxygen dependency at 1 year of age [42, 51]. The combination of o/e TFLV and o/e LHR was slightly more predictive of CLD than US assessment alone [44].

#### Liver position

It is now recognized that liver herniation itself and the subsequent lung volume restriction are independent risk factors for poor outcomes in patients with CDH (Table 1) [12, 52–55]. Conventionally, the liver position is classified as “up” (liver in the thorax) or “down” (liver in the abdomen). However, volumetric quantification of liver herniation predicts neonatal survival better than this dichotomous classification [18, 56–58]. The amount of intrathoracic parenchyma can be assessed by calculating either the liver-to-thoracic volume ratio (LiTR) or the liver herniation percentage (%LH).

**Fig. 2:**
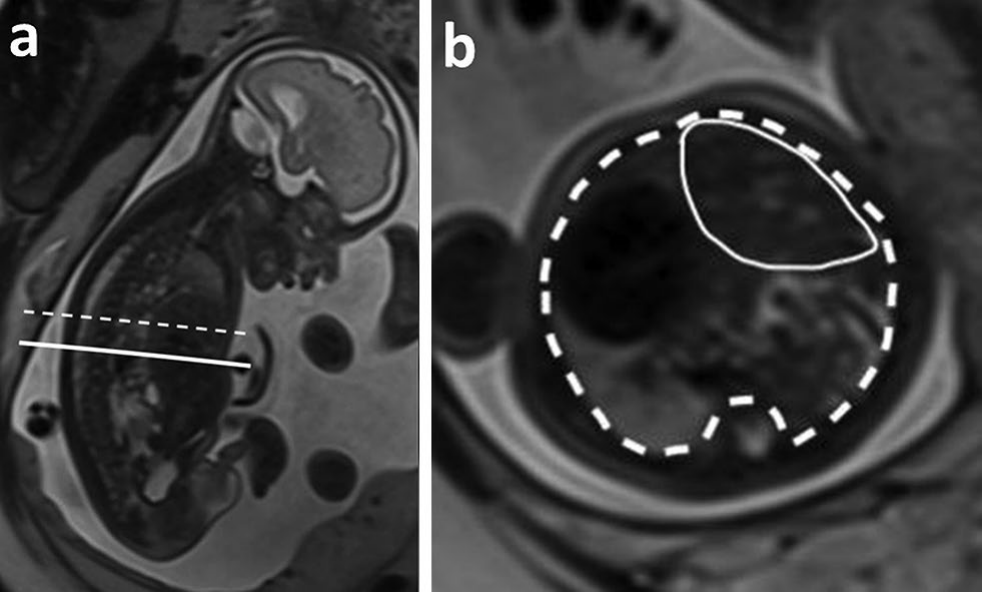
Liver-to-thoracic volume ratio (LiTR) calculation. **a** T2 HASTE sagittal image shows the line drawn at the xiphoid process (continuous line) for the first plane of measurement of the LiTR, and another line indicates a plane higher in the thorax at the liver top (dotted line). **b** T2 HASTE image in the axial plane shows the delineation of the liver (continuous line) and the thoracic cavity (dashed line). The LiTR is obtained by dividing the herniated liver volume by the total chest volume

LiTR is obtained by dividing the herniated liver volume by the total chest volume (Fig. 2). LiTR was found to be reproducible and to predict neonatal survival independently from lung volume in both right- and left-sided isolated CDH, with a suggested cutoff of 20% [54, 57]. Furthermore, the LiTR predicted survival in both expectantly managed CDH and those undergoing prenatal FETO treatment and was also predictive of post-FETO lung response [59]. Additionally, low LiTR was also associated with postnatal ECMO [56].

**Fig. 3:**
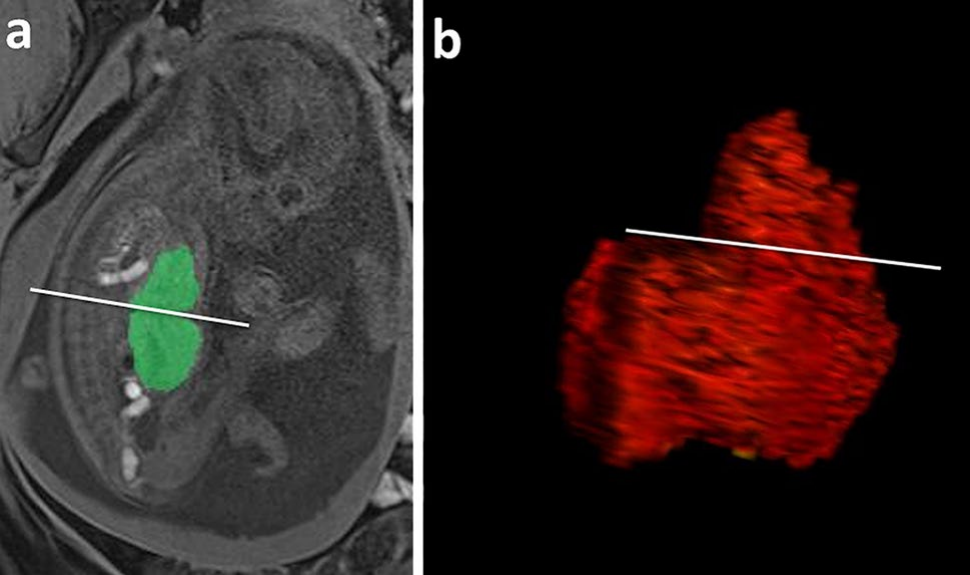
Percentage of liver herniation measurement (%LH). **a** T1 WIBE image in a sagittal view shows the liver segmentation methods with liver area measured on each slice (green) to obtain the 3D fetal level volume. **b** In both images, the white line drawn at the xiphoid process indicates the position of the diaphragm. The liver herniation percentage is then calculated by dividing the hepatic volume above the diaphragm by the entire liver volume

The %LH is calculated by dividing the hepatic volume above the diaphragm by the entire liver volume (Fig. 3). Studies demonstrated that the %LH was significantly lower in survivors and was associated with mortality and the need for ECMO, with the best cutoff value of 21% [35, 56]. In addition, Ruano et al. found that the combination of MRI measurement of total lung and liver herniation volumes was the most accurate in predicting neonatal mortality and ECMO’s need [58].

Liver herniation was shown to have an impact also on postnatal morbidity. Zamora et al. demonstrated that patients with CLD had a higher proportion of herniated liver than those without CLD. Liver herniation > 20% was independently associated with the need for oxygen supplementation at 30 days and showed an 11-fold higher likelihood of developing pulmonary sequelae. They concluded that the %LH represented the strongest predictor of CLD in patients with CDH [50].

#### Stomach position

The stomach can be easily recognized in the US and differentiated from other structures [8]. Grading systems for stomach position in US have been proposed to correlate with neonatal mortality [60, 61]. The four-step classification system of Kitano et al. evaluates the reciprocal position of the stomach and the heart on the coronal section of the thorax [60]. However, whether the stomach represents a genuinely independent risk factor has still to be proven [62]. Nawapun et al. defined the stomach to thorax ratio (STR) in left isolated CDH as the thoracic cavity volume occupied by the herniated stomach in fetal MRI. However, it did not show a correlation with the o/e TFLV. They also categorized the stomach position on MRI according to Kitano and al. and introduced an additional category in which the stomach was entirely dislocated on the contralateral side. They found an inverse relationship between o/e TFLV and the degree of stomach herniation [63].

#### Size of the defect

The size of the diaphragmatic defect significantly affects the surgical approach, especially regarding primary or patch repair [64–68]. In order to introduce an objective method of defect quantification, Rygl et al. proposed the perioperative calculation of the defect-diaphragmatic ratio (DDR) by dividing the area of the defect by the area of the diaphragm. They demonstrated that the DDR was objective and correlated well with primary repair’s feasibility [67].

Recently, Prayer et al. performed the first retrospective study to assess the validity of fetal MRI 3D reconstruction to locate, classify, and quantify diaphragmatic defects in 46 fetuses with CDH. They demonstrated that prenatal MRI 3D diaphragmatic segmentation is feasible, reproducible, and allows a correct identification and classification in all cases. They also calculated the DDR on fetal MRI and found that it was predictive of the need for patch repair, concluding that early MRI DDR evaluation could complement the existing parameters in prenatal counseling of fetuses with CDH [69].

#### Mediastinal shift angle

**Fig. 4:**
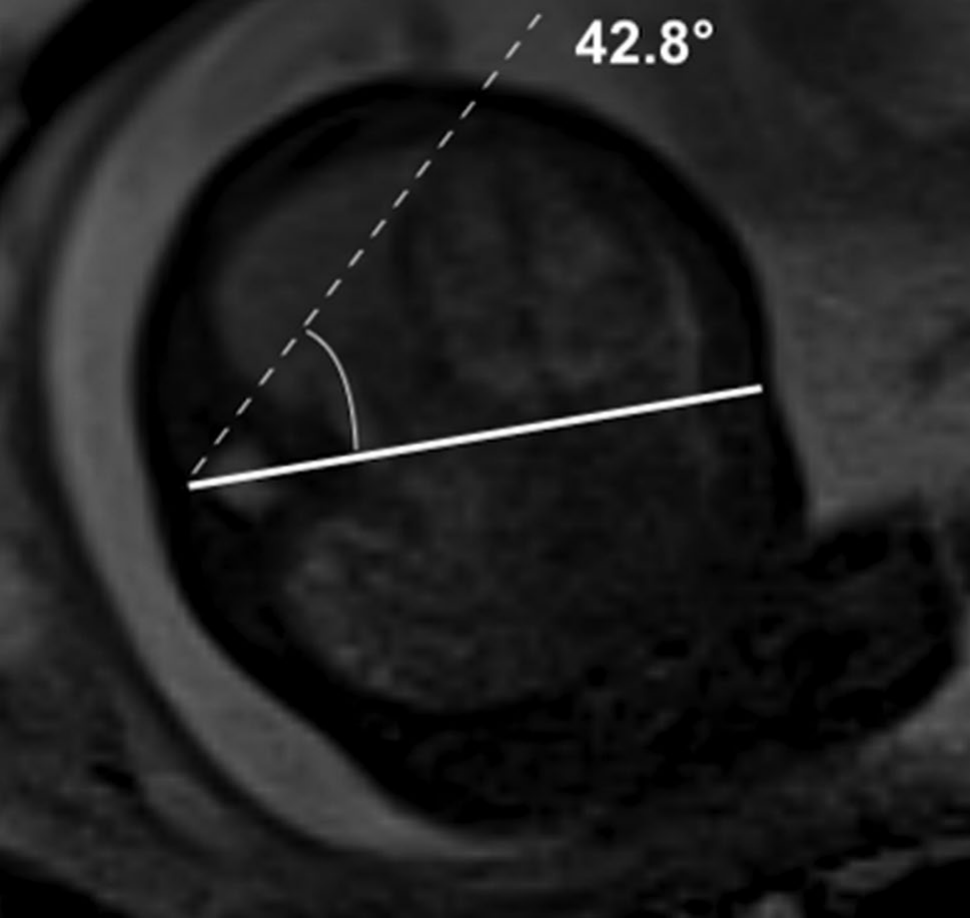
Mediastinal shift angle (MSA) calculation. True-Fisp axial image at the level of four-chamber view of the heart shows a sagittal midline (continuous line) drawn from the posterior face of the vertebral body to the mid of the sternum and a second line (dashed line) drawn from the same point of the vertebral body to touch the lateral wall of the right atrium tangentially

Volumetric assessment either through o/e LHR or o/e TFLV is operator-dependent, needs post-processing calculation, including a dedicated software for 3D reproduction of the lungs, and could be time-consuming. However, the mediastinal shift angle (MSA) has recently been proposed as a fast and reproducible measurement that could be calculated at US and MRI to assess hernia severity in isolated left-sided CDH [70–72]. The displacement of the mediastinal axis reflects the presence of herniated organs affecting the contralateral lung development. On fetal MRI, the MSA is obtained from an axial “true fast imaging with steady-state free precession” (TRUFI) at the level of a four-chamber view of the heart. First, a sagittal midline is drawn from the posterior face of the vertebral body to the mid of the sternum. Then, a second line is drawn from the same point of the vertebral body to touch the lateral wall of the right atrium tangentially (Fig. 4). The MSA was inversely related to TFLV and significantly lower in non-survivors than survivors [71, 72]. Among survivors, MSA increase was also associated with longer inotropic and vasoactive support, treatment with pulmonary vasodilators, mechanical ventilation, and length of stay [73]. However, data are limited and obtained from small cohorts of patients. Therefore, further investigation has to investigate whether the MSA could add value to the prognostic evaluation of fetuses with CDH.

### Other anatomical parameters

#### Hernia sac

In percentages ranging from 14.2 to 25.7% of CDH, the herniated organs are covered by a peritoneal layer forming a hernia sac [74, 75]. Several authors reported higher survival rates, more significant lung volumes, lower degree of liver herniation, and better postnatal outcomes in those patients with hernia sac, including the decreased need for ECMO and shorter mechanical ventilation [74–80]. In addition, when a sac constrains the hernia content, the diaphragmatic defect tends to be smaller and primary repair is usually feasible [50, 76, 79].

Zamora et al. described three MRI features that specifically correlated with the presence of hernia sac: (1) presence of lung meniscus posterior or apical to the hernia content; (2) encapsulated appearance of hernia contents, exerting more negligible mass effect on the heart and mediastinum than expected; and (3) presence of pleural fluid outlying a sac from above [81]. Prenatal identification of the sac could help in risk stratification and add useful information to the expected clinical course [7, 79].

#### Associated anomalies

CDH is associated with non-diaphragmatic congenital anomalies in almost half of the cases. In addition, these complex forms of CDH are characterized by a poorer prognosis. Therefore, prenatal screening for other malformations is mandatory [82, 83].

Cardiac malformations represent the most common associated anomalies, found in up to one-third of all CDH and 15% of non-syndromic cases [7]. Beyond structural alterations, there is increasing evidence that cardiac dysfunction contributes to CDH pathophysiology and poor postnatal outcome [84]. It has been shown that fetal left heart structures are smaller, regardless of the defect side, and left ventricular (LV) hypoplasia correlates with lung hypoplasia. Impaired embryological cardiac development, direct compression by herniated organs, reduced LV filling, and mediastinal shift contribute to fetal LV hypoplasia. However, the pathophysiological basis has not been fully clarified [84]. Prenatal US is routinely used to assess cardiac dysfunction in the fetal period, but cardiac MRI is beginning to spread also in this field. Recently, Yadav et al. suggested the use of fetal cine cardiac MRI using maternal electrocardiography for cardiac gating to calculate the right and left ventricular ejection fraction in a fetus with left CDH [85]. However, we are still a long way from the systematic MRI study of the fetal heart.

Although the real incidence is still unknown, some studies reported the presence of another concomitant lung lesion in up to 30% of CDH, such as congenital pulmonary airway malformation (CPAM), congenital cystic adenomatoid malformation (CCAM), and bronchopulmonary sequestration (BPS) [86–90]. However, little is known about how an associated congenital lung lesion affects postnatal outcomes, and the increasing application of fetal MRI could add further knowledge for these complex forms [87, 89].

### Pulmonary hypertension

Predicting the occurrence and severity of pulmonary hypertension (PH) is still the major challenge in prognostic evaluation of fetuses with CDH [7].

Lung hypoplasia is invariably associated with reduced vascular network extension, remodeling, and impaired vasoreactivity [91]. Although the pathogenesis of PH has not been yet fully clarified, the condition is believed to have a *fixed* morphological and a *reversible* functional component and involve alterations of endothelium, vascular smooth muscle cells, and altered vascular growth pathways [91–98].

PH plays a crucial role in mortality and long-term morbidity. Ideally, we should predict PH independently from pulmonary hypoplasia [5, 8]. Regrettably, lung volume is not always correlated with lung function and degree of PH [8]. Although lower o/e TFLV was associated with occurrence and severity of PH, it was also associated with higher mortality, with similar risk. Due to the close relationship between PH and mortality, it is difficult to determine the exact contribution of vascular development to the outcome by measuring lung volume. However, it could indirectly estimate vascular bed development [74]. A recent meta-analysis by Russo et al. showed that lung size and liver herniation predicted ECMO’s needs but not PH [5].

Direct assessment of lung vascularization represents the logical approach. Several attempts have been made to evaluate vascular development directly in utero through Doppler techniques, anatomical parameters, and vascular indices at 2D and 3D US, including hyperoxygenation tests. However, these measurements are difficult to reproduce, and their added predictive value remains uncertain [5, 62, 99–109].

Vuletin et al. evaluated the potential of prenatal predictors of severe postnatal PH in left isolated CDH using ultrafast fetal MRI. Diameters of the right pulmonary artery, left pulmonary artery, aorta, and length of the cerebellar vermis as reference measures were obtained to calculate two parameters: (1) prenatal PH index (PPHI) [PPHI = (left pulmonary artery/length of vermis) × 10)] and (2) McGoon index (MGI) [MGI = (right pulmonary artery + left pulmonary artery)/aorta] [110]. The authors demonstrated that PPHI and MGI negatively correlated with PH and were significantly lower in those patients with severe systemic/supra-systemic PH. Furthermore, they accurately predict PH at three weeks of life, with MGI the most sensitive predictor. There was no difference between survivors and non-survivors, and no relationship with mortality was found, probably due to the small sample size. In contrast, none of the standard parameters used to estimate lung volumes, such as LHR and TFLV, and predicted PH severity [110].

In conclusion, given the drastic hemodynamic changes occurring right after birth, in utero evaluation of PH is still challenging [36]. Therefore, as future direction, it is mandatory to consolidate other imaging methods to evaluate CDH-associated PH through non-volumetric parameters [24]. The availability of an alternative approach through the study of prenatal MRI and US images based on artificial intelligence, using machine and deep learning methods, could help develop a prenatal predictive PH model with high sensitivity and specificity in the next future [111].

### Qualitative evaluation

#### Diffusion-weighted MRI and apparent diffusion coefficient

Diffusion-weighted (DW) MRI assesses the translational mobility of water molecules in tissue when exposed to a magnetic field gradient [112]. DW MRI has been proposed as a qualitative method to evaluate microstructural characteristics of the developing lung [13, 113]. The apparent diffusion coefficient (ADC) is a quantitative-derived parameter that combines the capillary perfusion and water diffusion in the extravascular extracellular space [114].

Significant changes in ADC values have been observed throughout the gestation in normal fetal lungs, probably reflecting distal airways and pulmonary vasculature development. CDH fetuses deviate from this typical pattern due to structural alterations of the hypoplastic lungs [13]. However, the application of DWI in CDH lacks external validation and is therefore considered not reproducible [7]. This technique is also time-consuming and very sensitive to motion artifacts. For these reasons, it is not routinely used in a clinical setting [7, 13].

#### Signal intensity ratio

The relative signal intensity on T2 sequences has been proposed as a qualitative imaging parameter to assess fetal lung maturity [115–117]. However, to evaluate its relative changes, lung signal intensity needs a reference structure close to the lung and whose signal intensity remains stable throughout pregnancy [115, 118]. In addition, high signal intensity is associated with high fluid content in small airways and alveoli, while low intensity suggests lower fluid and lung immaturity [115]. Based on this knowledge, some studies have focused on signal intensity ratios in CDH patients as potential outcome predictors, but the results are controversial [31, 36, 118, 119].

Yamoto et al. calculated the lung-to-liver signal intensity ratio (LLSIR) using the contralateral lung region of interest analysis in isolated left-sided CDH compared to controls. LLSIR significantly increased during pregnancy in normal fetuses, while CDH fetuses did not, especially those with poor prognoses. They concluded that LLSIR was a marker of fetal lung maturity with promising prognostic applications, with o/e LLSIR cutoff value of 70 being the most accurate [118].

Dütemeyer et al. compared various signal intensity ratios on T2-weighted images to the o/e TFLV in predicting survival in isolated CDH. The LLSIR, lung-to-amniotic fluid signal intensity ratio (LAFSIR), lung-to-muscle signal intensity ratio (LMSIR), and lung-to-spinal fluid signal intensity ratio (LSFSIR) were calculated using region of interest (ROI) analysis both in the contralateral and ipsilateral lung. Among all signal intensity ratios, LLSIR correlated well with the prediction of postnatal survival. However, the o/e TFLV was by far superior in outcome prediction. Even if the developmental changes in signal intensity ratios were confirmed, qualitative evaluation through these parameters was less sensitive and less specific than quantitative analysis of lung maturity [36, 119]. Further investigations are required to clarify whether the combination of these parameters could improve outcome prediction in CDH.

## Experience and learning curve

Effective prenatal counseling largely relies on proper prognostic parameters on prenatal imaging. Both fetal US and MRI are operator-dependent and are often used in sequence to maximize the accuracy of the provided information [9, 38]. While the clinician could be relatively confident with the predictive accuracy for mild and extreme/severe cases, the broad spectrum of presentation of the intermediate forms poses additional challenges. For this reason, experience plays a key role as it could directly impact predictive value and the information we provide to the families [38]. In addition, prenatal imaging has a significant learning curve. Therefore, restricting the performance to a limited number of specialized operators could maximize expertise and predictive accuracy [36]. Finally, experience in managing CDH newborns needs to be considered as it is known to impact survival chances [120]. In light of these considerations, planning the birth of an affected fetus in a high-volume activity center where obstetrics, neonatologists, pediatric surgeons, radiologists, and nurses have achieved significant experience in the perinatal management of CDH improves neonatal outcomes [36].

## Future directions

Since MRI is expensive and unavailable in all centers, its use for primary diagnosis is still limited, and the quality across centers has not been tested extensively [9]. Despite the increasing use of fetal MRI during the last decade, prenatal US remains the most widely used tool in clinical practice [121]. However, MRI should be further encouraged in the prenatal workup due to its technical advantages, better anatomical definition, and superior FLV estimation and survival prediction. To maximize the potential of fetal MRI, standardization of indications, methodology, and data interpretation is required. This would provide consistent information for prenatal counseling, risk stratification, decision-making approach, and more comparable data across institutions [19]. Data integration from different imaging modalities remains the most desirable strategy [9].

In particular, the increasing application of MRI combined with the cardiac US will provide further knowledge of fetal heart dysfunction, which is now considered a key contributor to CDH pathophysiology, along with pulmonary hypoplasia and pulmonary hypertension [84, 85].

The growing interest in MRI is also moving towards its application in the postnatal period. Although our knowledge concerning prenatal lung growth in CDH patients has dramatically improved during the past decade, our understanding of postnatal lung growth is still limited. Therefore, MRI is increasingly being used to study the postoperative changes in newborns’ lung volumes with CDH, contributing to understanding lung catch-up growth [122]. It has been shown that increased lung volume progressively takes place after surgical repair and that the ipsilateral lung’s contribution to the total lung volume increase was even more significant in the most severe forms [122]. Recently, the feasibility of MRI for postnatal assessment of pulmonary vascularity in infants with CDH was investigated, showing a strong correlation with prenatal and postnatal markers of PH severity [123]. Although with limitations, this highlights the potential role of postnatal MRI for further understanding lung parenchymal and vascular hypoplasia, lung catch-up growth, and long-term pulmonary morbidity in infants with CDH.

Advances in technologies are opening doors to innovative MRI applications. Through additive manufacturing, Prayer et al. developed a 3D-printed life-sized model of the diaphragm, diaphragmatic defect, liver, and liver veins of a term CDH fetus, based on MRI acquisitions. A physical life-sized model derived from 3D segmentation data could help counsel the parents regarding such a complex malformation and support the pediatric surgeon in defining the optimal surgical strategy, including potentially identifying cases suitable for tissue-engineering-based treatment [69]. Regenerative tissue-engineering techniques may represent the future of personalized treatment in patients with CDH [124, 125]. In the future, fetal MRI may permit a 3D printable template for a prenatally available, ready-to-use, and tailor-made diaphragmatic patch obtained through regenerative tissue solutions [69].

## Conclusions

In the last years, huge progress has been made in the pre- and postnatal evaluation of fetuses with CDH [2, 7, 15, 73, 126, 127]. Fetal MRI has contributed chiefly to increasing our knowledge and predictive accuracy and will continue to strengthen its crucial role in the near future. However, none of the available markers accurately predict postnatal outcomes, particularly PH and long-term morbidity. In addition, several unexpected factors could step in the postnatal course to modify the final prognosis [8]. Therefore, imaging data must be combined with clinical variables and experience to perform a comprehensive prognostic assessment.

## Data Availability

N/A.

## Abbreviations

%LH: Liver herniation percentage
ADC: Apparent diffusion coefficient
BPS: Bronchopulmonary sequestration
CCAM: Congenital cystic adenomatoid malformation
CDH: Congenital diaphragmatic hernia
CLD: Chronic lung disease
CPAM: Congenital pulmonary airway malformation
DDR: Defect-diaphragmatic ratio
DWI: Diffusion-weighted images
ECMO: Extracorporeal membrane oxygenation
eFLV: Expected fetal lung volume
FFE: Fast field echo
FISP: True fast imaging with steady precession
FLASH: Fast low angle shot
HASTE: Half-Fourier single-shot turbo spin-echo
LHR: Lung-to-head ratio
LiTR: Liver-to-thoracic volume ratio
LLSIR: Lung-to-liver signal intensity ratio
MGI: McGoon Index
MRI: Magnetic resonance imaging
MSA: Mediastinal shift angle
NICU: Neonatal Intensive Care Unit
O/E LHR: Observed/expected lung-to-head ratio
O/E TFLV: Observed/expected total fetal lung volume
oFLV: Observed fetal lung volume
PH: Pulmonary hypertension
PPHI: Prenatal pulmonary hypertension index
PPLV: Percentage of predicted lung volume
ROI: Fetal region of interest
SSFP: Steady-state free precession
SS-FSE: Single-shot fast spin-echo
STR: Stomach to thoracic ratio
TFLV: Total fetal lung volume
TRUFI: True fast imaging with steady-state free precession
US: Ultrasound

## Code availability

N/A.

## References

[1] Rubesova E (2016). Why do we need more data on MR volumetric measurements of the fetal lung?. Pediatr Radiol.

[2] Russo FM, De Coppi P, Allegaert K, Toelen J, van der Veeken L, Attilakos G, Eastwood MP, David AL, Deprest J (2017). Current and future antenatal management of isolated congenital diaphragmatic hernia. Semin Fetal Neonatal Med.

[3] Snoek KG, Greenough A, Van Rosmalen J, Capolupo I, Schaible T, Ali K, Wijnen RM, Tibboel D (2018). Congenital diaphragmatic hernia: 10-year evaluation of survival, extracorporeal membrane oxygenation, and foetoscopic endotracheal occlusion in four high-volume centres. Neonatology.

[4] Coughlin MA, Werner NL, Gajarski R, Gadepalli S, Hirschl R, Barks J, Treadwell MC, Ladino-Torres M, Kreutzman J, Mychaliska GB (2016). Prenatally diagnosed severe CDH: mortality and morbidity remain high. J Pediatr Surg.

[5] Russo FM, Eastwood MP, Keijzer R, Al-Maary J, Toelen J, Van Mieghem T, Deprest JA (2017). Lung size and liver herniation predict need for extracorporeal membrane oxygenation but not pulmonary hypertension in isolated congenital diaphragmatic hernia: systematic review and meta-analysis. Ultrasound Obstet Gynecol.

[6] Jani J, Nicolaides KH, Keller RL, Benachi A, Peralta CF, Favre R, Moreno O, Tibboel D, Lipitz S, Eggink A, Vaast P, Allegaert K, Harrison M, Deprest J, Group A-C-R (2007). Observed to expected lung area to head circumference ratio in the prediction of survival in fetuses with isolated diaphragmatic hernia. Ultrasound Obstet Gynecol.

[7] Cordier A-G, Russo FM, Deprest J, Benachi A (2020). Prenatal diagnosis, imaging, and prognosis in congenital diaphragmatic hernia. Semin Perinatol.

[8] Benachi A, Cordier AG, Cannie M, Jani J (2014). Advances in prenatal diagnosis of congenital diaphragmatic hernia. Semin Fetal Neonatal Med.

[9] Kovler ML, Jelin EB (2019). Fetal intervention for congenital diaphragmatic hernia. Semin Pediatr Surg.

[10] Mehollin-Ray AR (2020). Congenital diaphragmatic hernia. Pediatr Radiol.

[11] Strizek B, Cos Sanchez T, Khalife J, Jani J, Cannie M (2015). Impact of operator experience on the variability of fetal lung volume estimation by 3D-ultrasound (VOCAL) and magnetic resonance imaging in fetuses with congenital diaphragmatic hernia. J Matern Fetal Neonatal Med.

[12] Oluyomi-Obi T, Kuret V, Puligandla P, Lodha A, Lee-Robertson H, Lee K, Somerset D, Johnson J, Ryan G (2017). Antenatal predictors of outcome in prenatally diagnosed congenital diaphragmatic hernia (CDH). J Pediatr Surg.

[13] Cannie M, Jani J, De Keyzer F, Roebben I, Dymarkowski S, Deprest J (2009). Diffusion-weighted MRI in lungs of normal fetuses and those with congenital diaphragmatic hernia. Ultrasound Obstet Gynecol.

[14] Mehollin-Ray AR, Cassady CI, Cass DL, Olutoye OO (2012). Fetal MR imaging of congenital diaphragmatic hernia. Radiographics.

[15] Dutemeyer V, Cordier AG, Cannie MM, Bevilacqua E, Huynh V, Houfflin-Debarge V, Verpillat P, Olivier C, Benachi A, Jani JC (2022). Prenatal prediction of postnatal survival in fetuses with congenital diaphragmatic hernia using MRI: lung volume measurement, signal intensity ratio, and effect of experience. J Matern Fetal Neonatal Med.

[16] Jani JC, Peralta CF, Ruano R, Benachi A, Done E, Nicolaides KH, Deprest JA (2007). Comparison of fetal lung area to head circumference ratio with lung volume in the prediction of postnatal outcome in diaphragmatic hernia. Ultrasound Obstet Gynecol.

[17] Jani J, Cannie M, Sonigo P, Robert Y, Moreno O, Benachi A, Vaast P, Gratacos E, Nicolaides KH, Deprest J (2008). Value of prenatal magnetic resonance imaging in the prediction of postnatal outcome in fetuses with diaphragmatic hernia. Ultrasound Obstet Gynecol.

[18] Bebbington M, Victoria T, Danzer E, Moldenhauer J, Khalek N, Johnson M, Hedrick H, Adzick NS (2014). Comparison of ultrasound and magnetic resonance imaging parameters in predicting survival in isolated left-sided congenital diaphragmatic hernia. Ultrasound Obstet Gynecol.

[19] Kim AG, Norwitz G, Karmakar M, Ladino-Torres M, Berman DR, Kreutzman J, Treadwell MC, Mychaliska GB, Perrone EE (2020). Discordant prenatal ultrasound and fetal MRI in CDH: wherein lies the truth?. J Pediatr Surg.

[20] Colleran GC, Kyncl M, Garel C, Cassart M (2022) Fetal magnetic resonance imaging at 3 Tesla - the European experience. Pediatr Radiol:1–12 10.1007/s00247-021-05267-6 35147713

[21] Saleem SN (2014). Fetal MRI: an approach to practice: a review. J Adv Res.

[22] Malamateniou C, Malik S, Counsell S, Allsop J, McGuinness A, Hayat T, Broadhouse K, Nunes R, Ederies A, Hajnal J (2013). Motion-compensation techniques in neonatal and fetal MR imaging. Am J Neuroradiol.

[23] Cassart M, Garel C (2020). European overview of current practice of fetal imaging by pediatric radiologists: a new task force is launched. Pediatr Radiol.

[24] Olutoye OA, Baker BW, Belfort MA, Olutoye OO (2018). Food and Drug Administration warning on anesthesia and brain development: implications for obstetric and fetal surgery. Am J Obstet Gynecol.

[25] Meyers ML, Mirsky DM, Dannull KA, Tong S, Crombleholme TM (2017). Effects of maternal valium administration on fetal MRI motion artifact: a comparison study at high altitude. Fetal Diagn Ther.

[26] Berger-Kulemann V, Brugger P, Pugash D, Krssak M, Weber M, Wielandner A, Prayer D (2013). MR spectroscopy of the fetal brain: is it possible without sedation?. Am J Neuroradiol.

[27] Chen Q, Levine D (2001). Fast fetal magnetic resonance imaging techniques. Top Magn Reson Imaging.

[28] Brugger PC, Stuhr F, Lindner C, Prayer D (2006). Methods of fetal MR: beyond T2-weighted imaging. Eur J Radiol.

[29] Rypens F, Metens T, Rocourt N, Sonigo P, Brunelle F, Quere MP, Guibaud L, Maugey-Laulom B, Durand C, Avni FE, Eurin D (2001). Fetal lung volume: estimation at MR imaging-initial results. Radiology.

[30] Gorincour G, Bouvenot J, Mourot MG, Sonigo P, Chaumoitre K, Garel C, Guibaud L, Rypens F, Avni F, Cassart M, Maugey-Laulom B, Bourliere-Najean B, Brunelle F, Durand C, Eurin D, Groupe Radiopediatrique de Recherche en Imagerie F (2005). Prenatal prognosis of congenital diaphragmatic hernia using magnetic resonance imaging measurement of fetal lung volume. Ultrasound Obstet Gynecol.

[31] Sebastia C, Garcia R, Gomez O, Pano B, Nicolau C (2014). Fetal magnetic resonance imaging evaluation of congenital diaphragmatic hernia. Radiologia (Roma).

[32] Ward VL, Nishino M, Hatabu H, Estroff JA, Barnewolt CE, Feldman HA, Levine D (2006). Fetal lung volume measurements: determination with MR imaging–effect of various factors. Radiology.

[33] Kolbe AB, Ibirogba ER, Thomas KB, Hull NC, Thacker PG, Hathcock M, Sangi-Haghpeykar H, Ruano R (2021). Reproducibility of lung and liver volume measurements on fetal magnetic resonance imaging in left-sided congenital diaphragmatic hernia. Fetal Diagn Ther.

[34] Meyers ML, Garcia JR, Blough KL, Zhang W, Cassady CI, Mehollin-Ray AR (2018). Fetal lung volumes by MRI: normal weekly values from 18 through 38 weeks’ gestation. AJR Am J Roentgenol.

[35] Victoria T, Bebbington MW, Danzer E, Flake AW, Johnson MP, Dinan D, Adzick NS, Hedrick HL (2012). Use of magnetic resonance imaging in prenatal prognosis of the fetus with isolated left congenital diaphragmatic hernia. Prenat Diagn.

[36] Dütemeyer V, Cordier AG, Cannie MM, Bevilacqua E, Huynh V, Houfflin-Debarge V, Verpillat P, Olivier C, Benachi A, Jani JC (2020) Prenatal prediction of postnatal survival in fetuses with congenital diaphragmatic hernia using MRI: lung volume measurement, signal intensity ratio, and effect of experience. J Matern Fetal Neonatal Med 1–9 10.1080/14767058.2020.1740982 32212880

[37] Jani J, Cannie M, Done E, Van Mieghem T, Van Schoubroeck D, Gucciardo L, Dymarkowski S, Deprest JA (2007). Relationship between lung area at ultrasound examination and lung volume assessment with magnetic resonance imaging in isolated congenital diaphragmatic hernia. Ultrasound Obstet Gynecol.

[38] Petroze RT, Caminsky NG, Trebichavsky J, Bouchard S, Le-Nguyen A, Laberge JM, Emil S, Puligandla PS (2019). Prenatal prediction of survival in congenital diaphragmatic hernia: an audit of postnatal outcomes. J Pediatr Surg.

[39] Neff KW, Kilian AK, Schaible T, Schutz EM, Busing KA (2007). Prediction of mortality and need for neonatal extracorporeal membrane oxygenation in fetuses with congenital diaphragmatic hernia: logistic regression analysis based on MRI fetal lung volume measurements. AJR Am J Roentgenol.

[40] Lee TC, Lim FY, Keswani SG, Frischer JS, Haberman B, Kingma PS, Habli M, Jaekle RK, Sharp G, Kline-Fath B, Rubio EI, Calvo M, Guimaraes C, Crombleholme TM (2011). Late gestation fetal magnetic resonance imaging-derived total lung volume predicts postnatal survival and need for extracorporeal membrane oxygenation support in isolated congenital diaphragmatic hernia. J Pediatr Surg.

[41] Alfaraj MA, Shah PS, Bohn D, Pantazi S, O’Brien K, Chiu PP, Gaiteiro R, Ryan G (2011). Congenital diaphragmatic hernia: lung-to-head ratio and lung volume for prediction of outcome. Am J Obstet Gynecol.

[42] Walleyo A, Debus A, Kehl S, Weiss C, Schönberg SO, Schaible T, Büsing KA, Neff KW (2013). Periodic MRI lung volume assessment in fetuses with congenital diaphragmatic hernia: prediction of survival, need for ECMO, and development of chronic lung disease. AJR Am J Roentgenol.

[43] Büsing KA, Kilian AK, Schaible T, Endler C, Schaffelder R, Neff KW (2008). MR relative fetal lung volume in congenital diaphragmatic hernia: survival and need for extracorporeal membrane oxygenation. Radiology.

[44] Schaible T, Büsing KA, Felix JF, Hop WC, Zahn K, Wessel L, Siemer J, Neff KW, Tibboel D, Reiss I, van den Hout L (2012). Prediction of chronic lung disease, survival and need for ECMO therapy in infants with congenital diaphragmatic hernia: additional value of fetal MRI measurements?. Eur J Radiol.

[45] Nawapun K, Sandaite I, DeKoninck P, Claus F, Richter J, De Catte L, Deprest J (2014). Comparison of matching by body volume or gestational age for calculation of observed to expected total lung volume in fetuses with isolated congenital diaphragmatic hernia. Ultrasound Obstet Gynecol.

[46] Cannie MM, Jani JC, Van Kerkhove F, Meerschaert J, De Keyzer F, Lewi L, Deprest JA, Dymarkowski S (2008). Fetal body volume at MR imaging to quantify total fetal lung volume: normal ranges. Radiology.

[47] Barnewolt CE, Kunisaki SM, Fauza DO, Nemes LP, Estroff JA, Jennings RW (2007). Percent predicted lung volumes as measured on fetal magnetic resonance imaging: a useful biometric parameter for risk stratification in congenital diaphragmatic hernia. J Pediatr Surg.

[48] Cannie M, Jani JC, Keyzer FD, Devlieger R, Schoubroeck DV, Witters I, Marchal G, Dymarkowski S, Deprest JA (2006). Fetal body volume: use at MR imaging to quantify relative lung volume in fetuses suspected of having pulmonary hypoplasia. Radiology.

[49] Debus A, Hagelstein C, Kilian AK, Weiss C, Schönberg SO, Schaible T, Neff KW, Büsing KA (2013). Fetal lung volume in congenital diaphragmatic hernia: association of prenatal MR imaging findings with postnatal chronic lung disease. Radiology.

[50] Zamora IJ, Olutoye OO, Cass DL, Fallon SC, Lazar DA, Cassady CI, Mehollin-Ray AR, Welty SE, Ruano R, Belfort MA, Lee TC (2014). Prenatal MRI fetal lung volumes and percent liver herniation predict pulmonary morbidity in congenital diaphragmatic hernia (CDH). J Pediatr Surg.

[51] Tsuda H, Kotani T, Miura M, Ito Y, Hirako S, Nakano T, Imai K, Kikkawa F (2017). Observed-to-expected MRI fetal lung volume can predict long-term lung morbidity in infants with congenital diaphragmatic hernia. J Matern Fetal Neonatal Med.

[52] Weis M, Hoffmann S, Henzler C, Weiss C, Schoenberg SO, Schaffelder R, Schaible T, Neff KW (2018). Isolated impact of liver herniation on outcome in fetuses with congenital diaphragmatic hernia—a matched-pair analysis based on fetal MRI relative lung volume. Eur J Radiol.

[53] Mayer S, Klaritsch P, Petersen S, Done E, Sandaite I, Till H, Claus F, Deprest JA (2011). The correlation between lung volume and liver herniation measurements by fetal MRI in isolated congenital diaphragmatic hernia: a systematic review and meta-analysis of observational studies. Prenat Diagn.

[54] Worley KC, Dashe JS, Barber RG, Megison SM, McIntire DD, Twickler DM (2009). Fetal magnetic resonance imaging in isolated diaphragmatic hernia: volume of herniated liver and neonatal outcome. Am J Obstet Gynecol.

[55] Khan AA, Furey EA, Bailey AA, Xi Y, Schindel DT, Santiago-Munoz PC, Twickler DM (2021). Fetal liver and lung volume index of neonatal survival with congenital diaphragmatic hernia. Pediatr Radiol.

[56] Lazar DA, Ruano R, Cass DL, Moise KJ, Johnson A, Lee TC, Cassady CI, Olutoye OO (2012). Defining “liver-up”: does the volume of liver herniation predict outcome for fetuses with isolated left-sided congenital diaphragmatic hernia?. J Pediatr Surg.

[57] Cannie M, Jani J, Chaffiotte C, Vaast P, Deruelle P, Houfflin-Debarge V, Dymarkowski S, Deprest J (2008). Quantification of intrathoracic liver herniation by magnetic resonance imaging and prediction of postnatal survival in fetuses with congenital diaphragmatic hernia. Ultrasound Obstet Gynecol.

[58] Ruano R, Lazar DA, Cass DL, Zamora IJ, Lee TC, Cassady CI, Mehollin-Ray A, Welty S, Fernandes CJ, Haeri S, Belfort MA, Olutoye OO (2014). Fetal lung volume and quantification of liver herniation by magnetic resonance imaging in isolated congenital diaphragmatic hernia. Ultrasound Obstet Gynecol.

[59] Cannie MM, Cordier AG, De Laveaucoupet J, Franchi-Abella S, Cagneaux M, Prodhomme O, Senat MV, Mokhtari M, Vlieghe V, Nowakowska D, Benachi A, Jani JC (2013). Liver-to-thoracic volume ratio: use at MR imaging to predict postnatal survival in fetuses with isolated congenital diaphragmatic hernia with or without prenatal tracheal occlusion. Eur Radiol.

[60] Kitano Y, Okuyama H, Saito M, Usui N, Morikawa N, Masumoto K, Takayasu H, Nakamura T, Ishikawa H, Kawataki M, Hayashi S, Inamura N, Nose K, Sago H (2011). Re-evaluation of stomach position as a simple prognostic factor in fetal left congenital diaphragmatic hernia: a multicenter survey in Japan. Ultrasound Obstet Gynecol.

[61] Cordier AG, Jani JC, Cannie MM, Rodo C, Fabietti I, Persico N, Saada J, Carreras E, Senat MV, Benachi A (2015). Stomach position in prediction of survival in left-sided congenital diaphragmatic hernia with or without fetoscopic endoluminal tracheal occlusion. Ultrasound Obstet Gynecol.

[62] Basurto D, Russo FM, Van der Veeken L, Van der Merwe J, Hooper S, Benachi A, De Bie F, Gomez O, Deprest J (2019). Prenatal diagnosis and management of congenital diaphragmatic hernia. Best Pract Res Clin Obstet Gynaecol.

[63] Nawapun K, Eastwood M, Sandaite I, DeKoninck P, Claus F, Richter J, Rayyan M, Deprest J (2015). Correlation of observed-to-expected total fetal lung volume with intrathoracic organ herniation on magnetic resonance imaging in fetuses with isolated left-sided congenital diaphragmatic hernia. Ultrasound Obstet Gynecol.

[64] Lally KP, Lally PA, Lasky RE, Tibboel D, Jaksic T, Wilson JM, Frenckner B, Van Meurs KP, Bohn DJ, Davis CF, Hirschl RB, Congenital Diaphragmatic Hernia Study G (2007). Defect size determines survival in infants with congenital diaphragmatic hernia. Pediatrics.

[65] Morini F, Valfre L, Capolupo I, Lally KP, Lally PA, Bagolan P, Congenital Diaphragmatic Hernia Study G (2013). Congenital diaphragmatic hernia: defect size correlates with developmental defect. J Pediatr Surg.

[66] Putnam LR, Gupta V, Tsao K, Davis CF, Lally PA, Lally KP, Harting MT, Group CDHS (2017). Factors associated with early recurrence after congenital diaphragmatic hernia repair. J Pediatr Surg.

[67] Rygl M, Kuklova P, Zemkova D, Slaby K, Pycha K, Stranak Z, Melichar J, Snajdauf J (2012). Defect-diaphragmatic ratio: a new parameter for assessment of defect size in neonates with congenital diaphragmatic hernia. Pediatr Surg Int.

[68] Macchini F, Raffaeli G, Amodeo I, Ichino M, Encinas JL, Martinez L, Wessel L, Cavallaro G (2022) Recurrence of congenital diaphragmatic hernia: risk factors, management, and future perspectives. Front Ped 10 10.3389/fped.2022.823180 PMC8864119 35223699

[69] Prayer F, Metzelder M, Krois W, Brugger PC, Gruber GM, Weber M, Scharrer A, Rokitansky A, Langs G, Prayer D, Unger E, Kasprian G (2019). Three-dimensional reconstruction of defects in congenital diaphragmatic hernia: a fetal MRI study. Ultrasound Obstet Gynecol.

[70] Romiti A, Viggiano M, Conforti A, Valfré L, Ravà L, Ciofi Degli Atti M, Bagolan P, Caforio L (2020). Ultrasonographic assessment of mediastinal shift angle (MSA) in isolated left congenital diaphragmatic hernia for the prediction of postnatal survival. J Matern Fetal Neonatal Med.

[71] Romiti A, Viggiano M, Savelli S, Salvi S, Vicario R, Vassallo C, Valfrè L, Tomà P, Bonito M, Lanzone A, Bagolan P, Caforio L (2020) Comparison of mediastinal shift angles obtained with ultrasound and magnetic resonance imaging in fetuses with isolated left sided congenital diaphragmatic hernia. J Matern Fetal Neonatal Med 1–6 10.1080/14767058.2020.1716714 31973612

[72] Savelli S, Bascetta S, Carducci C, Carnevale E, Caforio L, Romiti A, Tomà P (2020). Fetal MRI assessment of mediastinal shift angle in isolated left congenital diaphragmatic hernia: a new postnatal survival predictive tool?. Prenat Diagn.

[73] Amodeo I, Borzani I, Corsani G, Pesenti N, Raffaeli G, Macchini F, Condo V, Persico N, Ghirardello S, Colnaghi M, Mosca F, Cavallaro G (2022). Fetal MRI mediastinal shift angle and respiratory and cardiovascular pharmacological support in newborns with congenital diaphragmatic hernia. Eur J Pediatr.

[74] Spaggiari E, Stirnemann J, Bernard JP, De Saint BL, Beaudoin S, Ville Y (2013). Prognostic value of a hernia sac in congenital diaphragmatic hernia. Ultrasound Obstet Gynecol.

[75] Panda SS, Bajpai M, Srinivas M (2013). Presence of hernia sac in prediction of postoperative outcome in congenital diaphragmatic hernia. Indian Pediatr.

[76] Zamora IJ, Cass DL, Lee TC, Welty S, Cassady CI, Mehollin-Ray AR, Fallon SC, Ruano R, Belfort MA, Olutoye OO (2013). The presence of a hernia sac in congenital diaphragmatic hernia is associated with better fetal lung growth and outcomes. J Pediatr Surg.

[77] Grizelj R, Bojanic K, Vukovic J, Novak M, Weingarten TN, Schroeder DR, Sprung J (2017). Hernia sac presence portends better survivability of isolated congenital diaphragmatic hernia with “Liver-Up”. Am J Perinatol.

[78] Bouchghoul H, Marty O, Fouquet V, Cordier AG, Senat MV, Saada J, Mokhtari M, Le Sache N, Martinovic J, Benachi A (2018). Congenital diaphragmatic hernia has a better prognosis when associated with a hernia sac. Prenat Diagn.

[79] Oliver ER, DeBari SE, Adams SE, Didier RA, Horii SC, Victoria T, Hedrick HL, Adzick NS, Howell LJ, Moldenhauer JS, Coleman BG (2019). Congenital diaphragmatic hernia sacs: prenatal imaging and associated postnatal outcomes. Pediatr Radiol.

[80] Levesque M, Derraugh G, Schantz D, Morris MI, Shawyer A, Lum Min SA, Keijzer R (2019). The presence of a hernia sac in isolated congenital diaphragmatic hernia is associated with less disease severity: a retrospective cohort study. J Pediatr Surg.

[81] Zamora IJ, Mehollin-Ray AR, Sheikh F, Cassady CI, Williams JL, Lee TC, Ruano R, Cass DL, Zhang W, Olutoye OO (2015). Predictive value of MRI findings for the identification of a hernia sac in fetuses with congenital diaphragmatic hernia. Am J Roentgenol.

[82] Hidaka N, Ishii K, Mabuchi A, Yamashita A, Ota S, Sasahara J, Murata M, Mitsuda N (2015). Associated anomalies in congenital diaphragmatic hernia: perinatal characteristics and impact on postnatal survival. J Perinat Med.

[83] Montalva L, Lauriti G, Zani A (2019). Congenital heart disease associated with congenital diaphragmatic hernia: a systematic review on incidence, prenatal diagnosis, management, and outcome. J Pediatr Surg.

[84] Patel N, Massolo AC, Kipfmueller F (2020). Congenital diaphragmatic hernia-associated cardiac dysfunction. Semin Perinatol.

[85] Yadav T, Rajagopal R (2022). Functional assessment with fetal cardiac MRI in congenital diaphragmatic hernia. Radiology.

[86] Grethel EJ, Farrell J, Ball RH, Keller RL, Goldstein RB, Lee H, Farmer DL, Harrison MR, Nobuhara KK (2008). Does congenital diaphragmatic hernia associated with bronchopulmonary sequestration portend a better prognosis?. Fetal Diagn Ther.

[87] Cruz SM, Akinkuotu AC, Cass DL, Lee TC, Cassady CI, Mehollin-Ray AR, Ruano R, Welty SE, Olutoye OO (2016). Space occupying lesions in the presence of congenital diaphragmatic hernia. J Pediatr Surg.

[88] Diesen DL, Megison S (2014). Congenital diaphragmatic hernia with associated pulmonary sequestration. J Pediatr.

[89] Soni S, Moldenhauer JS, Rintoul N, Adzick NS, Hedrick HL, Khalek N (2020). Perinatal outcomes in fetuses prenatally diagnosed with congenital diaphragmatic hernia and concomitant lung lesions: a 10-year review. Fetal Diagn Ther.

[90] Aksoy Ozcan U, Altun E, Abbasoglu L (2012). Space occupying lesions in the fetal chest evaluated by MRI. Iran J Radiol.

[91] Sluiter I, van der Horst I, van der Voorn P, Boerema-de Munck A, Buscop-van Kempen M, de Krijger R, Tibboel D, Reiss I, Rottier RJ (2013). Premature differentiation of vascular smooth muscle cells in human congenital diaphragmatic hernia. Exp Mol Pathol.

[92] Pierro M, Thebaud B (2014). Understanding and treating pulmonary hypertension in congenital diaphragmatic hernia. Semin Fetal Neonatal Med.

[93] Alphonse RS, Vadivel A, Fung M, Shelley WC, Critser PJ, Ionescu L, O’Reilly M, Ohls RK, McConaghy S, Eaton F (2014). Existence, functional impairment, and lung repair potential of endothelial colony-forming cells in oxygen-induced arrested alveolar growth. Circulation.

[94] Acker SN, Seedorf GJ, Abman SH, Nozik-Grayck E, Partrick DA, Gien J (2013). Pulmonary artery endothelial cell dysfunction and decreased populations of highly proliferative endothelial cells in experimental congenital diaphragmatic hernia. American Journal of Physiology-Lung Cellular and Molecular Physiology.

[95] Acker SN, Mandell EW, Sims-Lucas S, Gien J, Abman SH, Galambos C (2015). Histologic identification of prominent intrapulmonary anastomotic vessels in severe congenital diaphragmatic hernia. J Pediatr.

[96] Baker CD, Black CP, Ryan SL, Balasubramaniam V, Abman SH (2013). Cord blood endothelial colony-forming cells from newborns with congenital diaphragmatic hernia. J Pediatr.

[97] Sbragia L, Nassr A, Gonçalves F, Schmidt A, Zuliani C, Garcia P, Gallindo R, Pereira L (2014). VEGF receptor expression decreases during lung development in congenital diaphragmatic hernia induced by nitrofen. Braz J Med Biol Res.

[98] Boucherat O, Franco-Montoya M-L, Delacourt C, Martinovic J, Masse V, Elie C, Thebaud B, Benachi A, Bourbon JR (2010). Defective angiogenesis in hypoplastic human fetal lungs correlates with nitric oxide synthase deficiency that occurs despite enhanced angiopoietin-2 and VEGF. American Journal of Physiology-Lung Cellular and Molecular Physiology.

[99] Cruz-Martinez R, Castanon M, Moreno-Alvarez O, Acosta-Rojas R, Martinez J, Gratacos E (2013). Usefulness of lung-to-head ratio and intrapulmonary arterial Doppler in predicting neonatal morbidity in fetuses with congenital diaphragmatic hernia treated with fetoscopic tracheal occlusion. Ultrasound Obstet Gynecol.

[100] Cruz-Martinez R, Martinez-Rodriguez M, Nieto-Castro B, Gamez-Varela A, Cruz-Lemini M, Luna-Garcia J, Juarez-Martinez I (2019). Longitudinal changes in lung size and intrapulmonary-artery Doppler during the second half of pregnancy in fetuses with congenital diaphragmatic hernia. Prenat Diagn.

[101] Moreno-Alvarez O, Hernandez-Andrade E, Oros D, Jani J, Deprest J, Gratacos E (2008). Association between intrapulmonary arterial Doppler parameters and degree of lung growth as measured by lung-to-head ratio in fetuses with congenital diaphragmatic hernia. Ultrasound Obstet Gynecol.

[102] Sokol J, Shimizu N, Bohn D, Doherty D, Ryan G, Hornberger LK (2006). Fetal pulmonary artery diameter measurements as a predictor of morbidity in antenatally diagnosed congenital diaphragmatic hernia: a prospective study. Am J Obstet Gynecol.

[103] Fuke S, Kanzaki T, Mu J, Wasada K, Takemura M, Mitsuda N, Murata Y (2003). Antenatal prediction of pulmonary hypoplasia by acceleration time/ejection time ratio of fetal pulmonary arteries by Doppler blood flow velocimetry. Am J Obstet Gynecol.

[104] Ruano R, Aubry M-C, Barthe B, Mitanchez D, Dumez Y, Benachi A (2006). Quantitative analysis of fetal pulmonary vasculature by 3-dimensional power Doppler ultrasonography in isolated congenital diaphragmatic hernia. Am J Obstet Gynecol.

[105] Hernandez-Andrade E, Thuring-Jönsson A, Jansson T, Lingman G, Maršál K (2004). Fractional moving blood volume estimation in the fetal lung using power Doppler ultrasound: a reproducibility study. Ultrasound in Obstetrics and Gynecology: the Official Journal of the International Society of Ultrasound in Obstetrics and Gynecology.

[106] DeKoninck P, Jimenez J, Russo FM, Hodges R, Gratacós E, Deprest J (2014). Assessment of pulmonary vascular reactivity to oxygen using fractional moving blood volume in fetuses with normal lung development and pulmonary hypoplasia in congenital diaphragmatic hernia. Prenat Diagn.

[107] Casaccia G, Crescenzi F, Dotta A, Capolupo I, Braguglia A, Danhaive O, Pasquini L, Bevilacqua M, Bagolan P, Corchia C, Orzalesi M (2006) Birth weight and McGoon Index predict mortality in newborn infants with congenital diaphragmatic hernia. J Pediatr Surg 41:25–28, discussion 25–28 10.1016/j.jpedsurg.2005.10.002 16410102

[108] Suda K, Bigras JL, Bohn D, Hornberger LK, McCrindle BW (2000). Echocardiographic predictors of outcome in newborns with congenital diaphragmatic hernia. Pediatrics.

[109] Takahashi S, Oishi Y, Ito N, Nanba Y, Tsukamoto K, Nakamura T, Ito Y, Hayashi S, Sago H, Kuroda T, Honna T (2009). Evaluating mortality and disease severity in congenital diaphragmatic hernia using the McGoon and pulmonary artery indices. J Pediatr Surg.

[110] Vuletin JF, Lim FY, Cnota J, Kline-Fath B, Salisbury S, Haberman B, Kingma P, Frischer J, Crombleholme T (2010). Prenatal pulmonary hypertension index: novel prenatal predictor of severe postnatal pulmonary artery hypertension in antenatally diagnosed congenital diaphragmatic hernia. J Pediatr Surg.

[111] Amodeo I, De Nunzio G, Raffaeli G, Borzani I, Griggio A, Conte L, Macchini F, Condo V, Persico N, Fabietti I, Ghirardello S, Pierro M, Tafuri B, Como G, Cascio D, Colnaghi M, Mosca F, Cavallaro G (2021). A maChine and deep Learning Approach to predict pulmoNary hyperteNsIon in newbornS with congenital diaphragmatic Hernia (CLANNISH): Protocol for a retrospective study. PLoS ONE.

[112] Luypaert R, Boujraf S, Sourbron S, Osteaux M (2001). Diffusion and perfusion MRI: basic physics. Eur J Radiol.

[113] Balassy C, Kasprian G, Brugger PC, Csapo B, Weber M, Hormann M, Bankier A, Bammer R, Herold CJ, Prayer D (2008). Diffusion-weighted MR imaging of the normal fetal lung. Eur Radiol.

[114] Le Bihan D, Breton E, Lallemand D, Aubin ML, Vignaud J, Laval-Jeantet M (1988). Separation of diffusion and perfusion in intravoxel incoherent motion MR imaging. Radiology.

[115] Kuwashima S, Nishimura G, Iimura F, Kohno T, Watanabe H, Kohno A, Fujioka M (2001). Low-intensity fetal lungs on MRI may suggest the diagnosis of pulmonary hypoplasia. Pediatr Radiol.

[116] Brewerton LJ, Chari RS, Liang Y, Bhargava R (2005). Fetal lung-to-liver signal intensity ratio at MR imaging: development of a normal scale and possible role in predicting pulmonary hypoplasia in utero. Radiology.

[117] Oka Y, Rahman M, Sasakura C, Waseda T, Watanabe Y, Fujii R, Makinoda S (2014). Prenatal diagnosis of fetal respiratory function: evaluation of fetal lung maturity using lung-to-liver signal intensity ratio at magnetic resonance imaging. Prenat Diagn.

[118] Yamoto M, Iwazaki T, Takeuchi K, Sano K, Fukumoto K, Takahashi T, Nomura A, Ooyama K, Sekioka A, Yamada Y, Urushihara N (2018). The fetal lung-to-liver signal intensity ratio on magnetic resonance imaging as a predictor of outcomes from isolated congenital diaphragmatic hernia. Pediatr Surg Int.

[119] Balassy C, Kasprian G, Brugger PC, Weber M, Csapo B, Herold C, Prayer D (2010). Assessment of lung development in isolated congenital diaphragmatic hernia using signal intensity ratios on fetal MR imaging. Eur Radiol.

[120] Grushka JR, Laberge JM, Puligandla P, Skarsgard ED, Canadian Pediatric Surgery N (2009). Effect of hospital case volume on outcome in congenital diaphragmatic hernia: the experience of the Canadian Pediatric Surgery Network. J Pediatr Surg.

[121] Van der Veeken L, Russo FM, De Catte L, Gratacos E, Benachi A, Ville Y, Nicolaides K, Berg C, Gardener G, Persico N (2018). Fetoscopic endoluminal tracheal occlusion and reestablishment of fetal airways for congenital diaphragmatic hernia. Gynecol Surg.

[122] Schopper MA, Walkup LL, Tkach JA, Higano NS, Lim FY, Haberman B, Woods JC, Kingma PS (2017). Evaluation of neonatal lung volume growth by pulmonary magnetic resonance imaging in patients with congenital diaphragmatic hernia. J Pediatr.

[123] Mukthapuram S, Beebe J, Tkach JA, Arya S, Haberman B, Peiro J, Lim F-Y, Woods JC, Kingma PS (2021). Magnetic resonance imaging assessment of pulmonary vascularity in infants with congenital diaphragmatic hernia: a novel tool for direct assessment of severity of pulmonary hypertension and hypoplasia. J Pediatr.

[124] De Coppi P, Deprest J (2017). Regenerative medicine solutions in congenital diaphragmatic hernia. Semin Pediatr Surg.

[125] Deprest J, Gucciardo L, Eastwood P, Zia S, Jimenez J, Russo F, Lesage F, Lewi L, Sampaolesi M, Toelen J (2014). Medical and regenerative solutions for congenital diaphragmatic hernia: a perinatal perspective. Eur J Pediatr Surg.

[126] Amodeo I, Pesenti N, Raffaeli G, Macchini F, Condo V, Borzani I, Persico N, Fabietti I, Bischetti G, Colli AM, Ghirardello S, Gangi S, Colnaghi M, Mosca F, Cavallaro G (2021) NeoAPACHE II. Relationship between radiographic pulmonary area and pulmonary hypertension, mortality, and hernia recurrence in newborns with CDH. Front Pediatr 9:692210 10.3389/fped.2021.692210 PMC8311172 34322463

[127] Amodeo I, Raffaeli G, Pesenti N, Macchini F, Condo V, Borzani I, Persico N, Fabietti I, Ophorst M, Ghirardello S, Gangi S, Colnaghi M, Mosca F, Cavallaro G (2020). The NeoAPACHE Study Protocol I: assessment of the radiographic pulmonary area and long-term respiratory function in newborns with congenital diaphragmatic hernia. Front Pediatr.

